# Built and socioeconomic environments: patterning and associations with physical activity in U.S. adolescents

**DOI:** 10.1186/1479-5868-7-45

**Published:** 2010-05-20

**Authors:** Janne Boone-Heinonen, Kelly R Evenson, Yan Song, Penny Gordon-Larsen

**Affiliations:** 1 Department of Nutrition, Gillings School of Global Public Health, University of North Carolina at Chapel Hill, North Carolina, USA; 2 Department of Epidemiology, Gillings School of Global Public Health, University of North Carolina at Chapel Hill, North Carolina, USA; 3 Department of City and Regional Planning, College of Arts and Sciences, University of North Carolina at Chapel Hill, North Carolina, USA

## Abstract

**Background:**

Inter-relationships among built and socioeconomic environmental characteristics may result in confounding of associations between environment exposure measures and health behaviors or outcomes, but traditional multivariate adjustment can be inappropriate due to collinearity.

**Methods:**

We used principal factor analysis to describe inter-relationships between a large set of Geographic Information System-derived built and socioeconomic environment measures for adolescents in the National Longitudinal Study of Adolescent Health (Wave I, 1995-96, n = 17,294). Using resulting factors in sex-stratified multivariate negative binomial regression models, we tested for confounding of associations between built and socioeconomic environment characteristics and moderate to vigorous physical activity (MVPA). Finally, we used knowledge gained from factor analysis to construct replicable environmental measures that account for inter-relationships and avoid collinearity.

**Results:**

Using factor analysis, we identified three built environment constructs [(1) homogenous landscape; 2) development intensity with high pay facility count; 3) development intensity with high public facility count] and two socioeconomic environment constructs [1) advantageous economic environment, 2) disadvantageous social environment]. In regression analysis, confounding of built environment-MVPA associations by socioeconomic environment factors was stronger than among built environment factors. In fully adjusted models, MVPA was negatively associated with the highest (versus lowest) quartile of homogenous land cover in males [exp(coeff) (95% CI): 0.91 (0.86, 0.96)] and intensity (pay facilities) [exp(coeff) (95% CI): 0.92 (0.85, 0.99)] in females. Single proxy measures (Simpson's diversity index, count of pay facilities, count of public facilities, median household income, and crime rate) representing each environmental construct replicated associations with MVPA.

**Conclusions:**

Environmental characteristics are inter-related. Both built and SES environments should be incorporated into analysis in order to minimize confounding. Single environmental measures may be useful proxies for environmental constructs in longitudinal analysis and replication in external populations, but more research is needed to better understand mechanisms of action, and ultimately identify policy-relevant environmental determinants of physical activity.

## Introduction

Numerous aspects of the built environment such as physical activity facilities (e.g., parks, recreation centers) [1,2], "walkability" [3,4], and neighborhood socioeconomic status (SES) [5-7] are related to physical activity and other key health behaviors and outcomes [8-10]. However, built and SES environments are theoretically and empirically correlated; for example, physical activity facilities are more common in wealthier neighborhoods [11] and streets may be more connected in the poor inner-city [12]. Therefore, neighborhood health studies that examine single or narrow sets of environmental characteristics are vulnerable to confounding by other environmental variables.

Strong correlations among environmental measures may also result in collinearity, thus precluding extensive covariate adjustment. Pattern analysis techniques such as factor analysis is a common strategy for overcoming collinearity and accounting for potentially interactive effects of environmental characteristics [12-16], but are limited in that they are data-driven and population specific. Further, extant replicable "walkability" and "urban sprawl" indices [17,18] do not incorporate other potentially important environmental features such as facilities [2,11]. Finally, most work has been in constrained geographic areas [18] or has used large geographic units such as counties [17].

While correlations between neighborhood SES and built environment characteristics may result from complex and dynamic relationships, they may also reflect independent clustering of characteristics in space. For example, a suburban neighborhood may exhibit low street connectivity and higher SES, but low street connectivity does not necessarily result from having more social and financial resources. Therefore, we conceptualize the built and SES environments as independent influences on physical activity, which allows comparison of built and SES environments and separation of more modifiable built environment from less modifiable SES environment factors.

Using nationally representative data on US adolescents, a group at risk for dramatic decline in physical activity [19,20], we sought to: (1) describe inter-relationships between a large set of built and SES environment measures in a nationally representative sample of adolescents, (2) quantify the extent to which inter-related environment measures confound associations with moderate to vigorous physical activity (MVPA), and (3) demonstrate a strategy for using pattern analysis results to construct replicable environment measures that accounts for inter-relationships and avoids collinearity.

## Methods

### Study population and data sources

We used cross-sectional Wave I data from The National Longitudinal Study of Adolescent Health (Add Health), a cohort study of 20,745 adolescents representative of the U.S. school-based population in grades 7 to 12 (11-22 years of age) in 1994-95. Add Health included a core sample plus subsamples of selected minority and other groupings collected under protocols approved by the Institutional Review Board at the University of North Carolina at Chapel Hill. The survey design and sampling frame are described elsewhere [21].

Neighborhood-level variables were created using a Geographic Information System (GIS) that links community-level data to Add Health respondent residential locations in space and time. Residential locations for adolescents in the probability sample (n = 18,924) were determined from the following sources, in order of priority: (1) geocoded home addresses with street-segment matches (n = 15,480), (2) global positioning system (GPS) measurements (n = 2,996), (3) ZIP/ZIP+4/ZIP+2 centroid match (n = 205), (4) respondent's geocoded school location (n = 243). Residential locations were linked to attributes of the circular area within 1 and 3 kilometers (k) of each respondent residence (Euclidean neighborhood buffer), block group, tract, and county attributes from U.S. Census and other federal sources, and Add Health survey data.

To facilitate national representation of adolescent neighborhood environments, missing environmental data (n = 630, 3.3%) was the only exclusion criterion for environmental patterning analyses, resulting in 18,294 adolescents. In estimating associations with MVPA, exclusions included self-reported pregnancy (n = 401) or mobility disability (n = 122) and Native Americans due to small sample size (n = 156); of the remaining sample (18,248), those with missing analytic variables (n = 359 missing individual-level variables, 578 missing environmental variables, 17 missing both) were also excluded for an analytical sample of 17,294 adolescents.

### Study variables

#### GIS-derived environmental characteristics

We examined built and SES environment measures with conceptual relevance or evidence of physical activity relationships in existing literature; see Table 1 for variable definitions and data sources and additional details below. We defined neighborhoods (e.g., 1 or 3 k buffer, or Census tracts) consistent with the strongest associations with MVPA in prior analysis (Boone-Heinonen J, Gordon-Larsen P, Song Y, Popkin BM: What is the relevant neighborhood area for detecting built environment relationships with physical activity?, submitted).

**Table 1:** Built and socioeconomic environment measures:^1 ^data sources and variable descriptions

Data source (year); Measure	**Geographic Area**^**2**^	Variable description
**ESRI StreetMap (2000)**		
Street connectivity		
Alpha index^3^	1 k	Ratio of observed to maximum possible route alternatives between nodes (intersections); high values indicate high connectivity.
Beta index^3^	1 k	Ratio of links (connections between nodes) to nodes; high values indicate high connectivity.
Cul de sac density^3^	1 k	Number of cul de sacs (single-link nodes) per square kilometer; low values indicate high connectivity.
Cyclomatic index^3^	1 k	Number of route alternatives between nodes; high values indicate high connectivity.
Gamma index^3^	1 k	Ratio of observed links to the maximum number of links; high values indicate high connectivity.
Intersection density	1 k	Number of 3- or more-way intersections (≥links in a single node) per square kilometer
**Commercial database of U.S. businesses (1995)**		
Physical activity facilities		
Instruction (count)	3 k	Dance studios, basketball instruction, martial arts
Member (count)	3 k	Athletic club and gymnasium, tennis club, basketball club
Outdoor (count)	3 k	Sporting and recreation camps, swimming pools
Public (count)	3 k	Public beach, pools, tennis courts, recreation centers
Public fee (count)	3 k	Physical fitness facilities, bicycle rental, public golf courses
Youth organization (count)	3 k	Boy/Girl Scouts, youth centers
**ESRI StreetMap Pro, parks component (2003)**		
Parks (count)	3 k	Local parks and recreation areas, classified by Census Bureau classification code
**National land cover dataset (1992)**		
Landscape diversity		
Mean patch size	1 k	Total land patch area divided by the number of patches (square meters)
Patch size variability	1 k	Square root of the sum of the squared deviations of each patch area from the mean patch area, divided by the number of patches
Land patch density	1 k	Number of land patches per hectare
Simpson's diversity index	1 k	Represents the probability that any two pixels selected at random would be different patch types.
Contagion index	1 k	Measures texture based on aggregation and interspersion of land patch types
Perimeter-fractal dimension	1 k	Measures perimeter and shape complexity
Patch richness^3^	1 k	Number of different patch types (classes)
Mean shape index^3^	1 k	Mean shape index, which measures patch shape and compaction.
Mean fractal dimension index^3^	1 k	Measures perimeter and shape complexity across a range of spatial scales (patch sizes)
**U.S. Census (1990)**		
Population count	1 k	Count of persons within buffer
% below poverty	CT	Percent of persons living in households with income below the federal poverty level
% minority	CT	Percent of persons with race/ethnicity other than white non-Hispanic
% college educated	CT	Percent of persons 25 years and older with at least a college education
Median household income	CT	Median household income
% homeowners	CT	Percent of households who own (versus rent) their homes
**Uniform Crime Reporting data (1995)**		
Crime rate	Co	Number of non-violent and violent crimes per 100,000 population

^1^From the National Longitudinal Study of Adolescent Health Obesity Environment Database

^2^1 k, 3 k = 1 and 3 kilometer Euclidean buffer; CT = census tract; Co = County. Selected neighborhood definitions were selected because they yielded the strongest associations between environment measures and physical activity in previous analysis.

^3^Examined in exploratory factor analysis but excluded from final factor solutions based on criteria described in Methods

We obtained ***PA facility counts ***from a historical dataset of U.S. businesses with high overall agreement between commercial and field data [22] and classified according to 8-digit Standard Industrial Classification codes into overlapping types (Table 1). Measures of ***landscape diversity and complexity ***[23] were created from national land cover data using Fragstats software [24]. Using classical graph theory [25], we created ***street connectivity ***measures reflecting the number and directness of route options [26]. We classified ***population density ***using census population counts within 1 k buffers weighted according to the proportion of the block-group area captured within 1 k, divided by buffer area.

SES environment measures included economic (median household income; proportion of persons below poverty, college degree or greater) and social (crime rate; proportion minority race/ethnicity, owning their homes) environment characteristics.

#### Individual-level self-reported behaviors and sociodemographics

MVPA was ascertained using a standard, interview administered, 7-item activity recall based on questionnaires validated in other epidemiologic studies [27-29]. Three items corresponding with MVPA (skating & cycling, exercise, and active sports) were summed to yield total weekly frequency (bouts) of MVPA. Individual-level sociodemographic control variables included age at Wave I interview, self-identified race (white, black, Asian, Hispanic), parent-reported annual household income and highest level of education (<high school, high school or GED, some college, ≥college degree), and administratively determined U.S. region (West, Midwest, South, Northeast). Distributions of these variables in the analytical sample are reported in the Appendix (see Table A1; additional file 1).

### Statistical analysis

#### Exploratory Factor Analysis (EFA)

We used EFA to describe inter-relationships across a large set of built and SES environment characteristics (Table 1). We used the principal factors estimator because it did not impose distributional constraints, oblique rotation (oblimin, gamma = 0) because environmental constructs are theoretically and empirically correlated, and Kaiser Criterion (Eigenvalue > 1), scree plots, and interpretability to determine the number of factors. Variables with weak loadings (<0.4) on all factors and variables of interest with substantial cross-loadings (>0.3) were removed from the EFA model. If two or fewer variables loaded strongly on a single factor, corresponding variables were removed from analysis. To address negative Eigenvalues, percent variance explained by each factor was calculated using the trace of the correlation matrix as the divisor [30]. EFA of SES environment variables was conducted separately using the same procedure.

#### Regression analysis

We fit two sets of regression models to estimate the relationships (1) among the resulting built and SES environment factors and (2) between the built and SES environment factors and MVPA. Because street connectivity measures did not load onto factors but were relevant to our analysis, we examined one index (alpha) as a single variable in our models that was not highly correlated with the built environment factors.

Buffer-based measures are individual-level variables. While census tracts and counties could comprise a second level in multi-level analysis, they are not nested within schools, the primary sampling unit and a more important source of clustering. Additionally, our data were sparse and unbalanced (mean = 8, range = 1-275 respondents per census tract), so multilevel analysis may have produced biased estimates [31]. Intraclass correlations for ln(MVPA) were minimal (0.03; ICC's are not definable for Poisson distributed outcomes). We therefore used single-level regression models, which corrected for complex survey sampling and were weighted for national representation.

We conducted all statistical analyses in Stata version 10.1. First, in a descriptive analysis examining the association between built and SES environments, we used crude linear regression to model each built environment factor and street connectivity (alpha index) as a function of SES environment factor quartiles. Second, to investigate confounding of built environment-MVPA associations by other environment variables, we fit a series of negative binomial regression models estimating weekly MVPA bouts as a function of built environment factor quartiles, controlling for cumulative sets of variables in Models 1-3: Model 1 included individual-level sociodemographic variables and one built environment factor or alpha; Model 2 added all three built environment factors and alpha; Model 3 added a 1-dimensional SES environment factor. In Model 4, a 2-dimensional SES environment construct replaced the 1-dimensional factor. Models were sex-stratified due to sex differences in physical activity determinants [32].

To account for non-linear relationships, we examined quartiles of the environment factors and measures. In contrast to continuous variables with higher-order terms, quartiles facilitated comparability with parallel analysis using single measures representing each factor (described below). For interpretability, results are reported as exponentiated coefficients, representing the proportion increase in MVPA bouts compared to the lowest quartile.

Confounding was objectively defined using a >±20% percent change in coefficient criterion [100*(current model-previous model)/previous model]; Models 3 and 4 were compared to Model 2. Because large percent changes reflect negligible absolute changes when coefficients are very small, our confounding definition was more stringent than the conventional 10% change threshold. Additionally, percent change was not reported if coefficients remained within ±0.04, corresponding to the approximate magnitude of marginally statistically significant coefficients.

As suggested by Riitters and colleagues [33], we evaluated single environmental measures (guided by strength of factor loadings and conceptual considerations) that could potentially serve as proxies for their respective constructs by replicating Models 1-4 above using single measures representing each factor. We selected Simpson's diversity index to represent the homogenous landscape factor. For the intensity factors, the counts of each type of facility were unstable, so non-overlapping pay facility types (instruction, member, and public fee) were summed. For public facilities, public (rather than youth) facilities were selected due to relevance across age groups. Because the preceding analyses suggested that resource counts represented general density of development, we separated the availability of resources from density by using alternative facilities variables calculated as the number of facilities per 1,000 population in Model 5.

## Results

### Patterning of the built and socioeconomic environments

The variability of built and SES environment measures included in the final factor solutions and subsequent analyses (Table 2) demonstrate the geographic diversity of the Add Health population. Percentile values and larger mean (versus median) values illustrate the right-skewed distribution of many measures.

**Table 2:** Built and socioeconomic environment characteristics: descriptive statistics^1^

Measure	mean (SE)	min	25th	median	75th	max
Street connectivity						
Alpha index	0.32 (0.01)	-8	0.22	0.30	0.38	8
Intersection density	29.5 (1.65)	0	8.6	26.4	44.6	168.1
Population density	1,410 (182)	0.05	128	794	1,779	29,961
Landscape diversity						
Mean patch size	30,196 (1,566)	7,411	14,213	88,250	32	315,000
Patch size variability	171,517 (6,853)	21,151	19,147	131,745	52	939,810
Land patch density	52.4 (1.7)	3.2	32.5	52.2	70.4	134.9
Simpson's diversity index	0.54 (0.01)	0.01	0.45	0.58	0.67	0.83
Contagion index	48.3 (0.9)	9.1	36.2	45.8	57.9	98.3
Perimeter-fractal dimension	1.48 (0.01)	1.06	1.44	1.50	1.54	1.67
Physical activity facilities						
Instruction	2.3 (0.2)	0	0	1	3	100
Member	2.0 (0.2)	0	0	1	3	52
Outdoor	1.2 (0.1)	0	0	1	2	15
Public	0.9 (0.1)	0	0	0	1	22
Public fee	1.4 (0.1)	0	0	1	2	22
Youth organization	1.3 (0.2)	0	0	0	2	38
Parks	4.7 (0.5)	0	0	1	8	44
Census measures						
% below poverty	0.15 (0.01)	0	0.06	0.11	0.21	0.85
% ≥ college education	0.23 (0.01)	0.01	0.13	0.19	0.29	0.82
% minority	0.21 (0.02)	0	0.02	0.08	0.29	1
Median household income	29,753 (935)	4,999	21,003	28,462	35,643	125,053
% homeowners	0.68 (0.01)	0	0.56	0.72	0.83	0.98
Crime rate	5,476 (237)	108	3,523	5,528	6,975	13,723

^1 ^National Longitudinal Study of Adolescent Health, Wave I (1995-96), n = 18,294. Excludes built environment characteristics examined but not included in subsequent analysis

Built environment measures were inter-correlated, loading onto three factors explaining 70.9% of variation (Table 3). Landscape variables loaded onto a single *homogeneous landscape *factor (high scores indicate non-diverse landscape) and two development *intensity *factors representing the degree of high intersection and population density and counts of either pay or public physical activity facilities (high scores indicate high development intensity). Conceptually, we expected correlation among facilities counts and density variables, so population and intersection density were retained despite cross-loadings. Unweighted correlations with *homogeneous landscape *were -0.03 and -0.02 for *intensity (pay facilities) *and *intensity (public facilities)*, respectively; and 0.58 between the two *intensity *factors. Other street connectivity indices did not load onto any factors and were therefore removed from factor analysis.

**Table 3:** Built environment factor loadings resulting from exploratory factor analysis^1^

	Homogenous landscape	Intensity (pay facilities)	Intensity (public facilities)
Patch size variability	**0.98**	--	--
Contagion index	**0.90**	--	--
Simpson's diversity index	**-0.90**^**2**^	--	--
Mean patch size	**0.87**	--	--
Land patch density	**-0.82**	--	--
Perimeter-fractal dimension	**-0.81**	--	--
Intersection density	-0.20	**0.48**	0.33
Population density	--	**0.41**	**0.50**
Facilities - instruction	--	**0.82**^**2**^	--
Facilities - member	--	**0.81**^**2**^	--
Facilities - outdoor	--	**0.80**	--
Facilities - public fee	--	**0.73**^**2**^	--
Facilities - public	--	--	**0.96**^**2**^
Facilities - youth organization	--	--	**0.90**^**2**^
Parks	--	0.40	0.34

^1 ^National Longitudinal Study of Adolescent Health, Wave I (1995-96), n = 18,294. Obtained from exploratory factor analysis using a principal factors estimator and oblique oblimin (gamma = 0) rotation. Unweighted correlations with *non-diverse landscape *were -0.03 and -0.02 for *intensity (pay facilities) *and *intensity (public facilities)*, respectively; and 0.58 between *intensity (pay facilities) *and *intensity (public facilities)*.

^2 ^Indicator variable(s) used to represent corresponding factor in **Tables 8 & 9**.

--For clarity, loadings with absolute value <0.2 were omitted

Two SES environment factors (Table 4) (unweighted correlation -0.49; 55.7% of variation explained) were consistent with our theorized constructs: one represented *advantageous economic environment *(high scores indicate low poverty, high college and median household income), the other represented characteristics typically associated with less desirable health outcomes (*disadvantageous social environment*; high scores indicate high proportion of racial/ethnic minorities and renters and high crime). Because the second factor marginally met inclusion criteria (Eigenvalue = 0.83), a 1-dimensional SES factor was also examined (41.9% of variation explained).

**Table 4:** Socioeconomic (SES) environment factor loadings resulting from exploratory factor analysis^1^

	2 factor solution		Alternative: 1 factor solution
	Advantageous economic environment	**Disadvantageous social environment **^**2**^	Advantageous socioeconomic environment
Median household income	**0.86**^3^	--	**0.85**
% ≥ college education	**0.84**	--	**0.62**
% below poverty	**-0.58**	0.46	**-0.87**
% minority	--	**0.61**	**-0.48**
Crime rate	--	**0.62**^3^	-0.30
% homeowners	--	**-0.56**	**0.57**

^1 ^National Longitudinal Study of Adolescent Health, Wave I (1995-96), n = 18,294. Obtained from exploratory factor analysis using a principal factors estimator and oblique oblimin (gamma = 0) rotation. Unweighted correlations between *Advantageous economic environment *and *Disadvantageous social environment *factors was -0.49

^2 ^Marginally met inclusion criteria (Eigenvalue = 0.83)

^3 ^Indicator variable(s) used to represent corresponding factor in **Tables 8 & 9**.

--For clarity, loadings with absolute value <0.2 were omitted

### Relationship between built and SES environments

Using factor scores generated from factor analysis, we examined built environment constructs within quartiles of the 2-dimensional SES environment constructs. Built environment factors or alpha street connectivity index (analyzed as a single variable because street connectivity variables were not derived into the final factor solution) varied across quartiles of SES factors (Table 5). The two SES environment factors were inversely related, but positively associated with the intensity factors. SES factors were negatively associated with less homogeneous landscape, whereas the disadvantageous social environment factor was positively associated with connectivity.

**Table 5:** Crude associations between built environment factor scores and socioeconomic environment factor quartiles [coeff (95% CI)]^1^

	Homogenous landscape	Intensity (pay facilities)	Intensity (public facilities)	Connectivity (alpha)
Advantageous economic environment score quartile				
1^2^	0	0	0	0
2	-0.29 (-0.63, 0.04)	0.46 (0.28, 0.64)*	0.20 (0.02, 0.37)*	-0.02 (-0.09, 0.05)
3	-0.50 (-0.86, -0.13)*	0.70 (0.46, 0.94)*	0.33 (0.11, 0.55)*	-0.01 (-0.10, 0.07)
4	-0.63 (-1.01, -0.25)*	0.90 (0.63, 1.18)*	0.40 (0.19, 0.61)*	-0.07 (-0.15, 0.00)
Disadvantageous social environment score quartile				
1^2^	0	0	0	0
2	0.10 (-0.18, 0.37)	0.29 (0.17, 0.40)*	0.31 (0.20, 0.42)*	0.08 (0.02, 0.15)*
3	-0.20 (-0.46, 0.05)*	0.62 (0.37, 0.87)*	0.57 (0.41, 0.73)*	0.06 (0.00, 0.12)*
4	-0.27 (-0.66, 0.12)*	1.25 (0.94, 1.55)*	1.33 (1.00, 1.66)*	0.09 (0.01, 0.16)*

^1^National Longitudinal Study of Adolescent Health, Wave I (1995-96), n = 18,294. Based on linear regression modeling each built environment factor from **Table 3 **(or street connectivity variable) as a function of quartiles of Advantageous economic and Disadvantageous social environment factor scores **(Table 4)**.

^2 ^Referent category is lowest quartile.

*Statistically significant (p < 0.05)

### MVPA and built and SES environment *factor scores*: associations and confounding

Next, we examined MVPA as a function of built and SES environment factor scores. By sequentially adjusting for additional variables in Models 1 through 4, we tested for confounding by different sets of environmental characteristics, quantified by the percent change in coefficients. In males, models adjusted for individual-level sociodemographics (Table 6, Model 1) showed that weekly MVPA bouts were 8% lower for males living in areas with the highest versus lowest landscape homogeneity score quartile. Analogous results for females showed relationships counter to theory: compared to the lowest quartiles, the highest intensity (pay facilities) and alpha street connectivity index quartiles were associated with 7% and 8% lower MVPA bouts, respectively (Table 7, Model 1). While inclusion of all four built environment measures indicated confounding by other built environment features according to our objective definition, the absolute change in estimates were small (Tables 6 &7, Model 1 vs. 2).

**Table 6:** Assessment of confounding to associations between built and socioeconomic environment **factor score quartiles **and weekly bouts of MVPA [exp(coeff)]^1^, Males (n = 8,668)

	**exp(coefficient) (95% CI) [change in coefficient**^**2**^**]**			
Quartile [median (range)]	Model 1	Model 2	Model 3	Model 4
Homogenous landscape score				
1 [-0.86 (-1.43, -0.68)]	1	1	1	1
2 [-0.49 (-0.68, -0.27)]	0.98 (0.93, 1.03)	0.98 (0.93, 1.03)	0.98 (0.93, 1.03)	0.98 (0.93, 1.03)
3 [0.00 (-0.27, 0.35)]	0.95 (0.91, 1.00)	0.95 (0.91, 1.00) [0%]	0.95 (0.91, 1.00)* [4%]	0.95 (0.91, 1.00)* [2%]
4 [1.04 (0.35, 5.46)]	0.92 (0.87, 0.97)*	0.90 (0.85, 0.95)* [18%]	0.90 (0.85, 0.96)* [-3%]	0.91 (0.86, 0.96)* [-5%]
Intensity (pay facilities) score				
1 [-0.82 (-1.45, -0.68)]	1	1	1	1
2[-0.51 (-0.68, -0.25)]	1.04 (1.00, 1.09)	1.02 (0.97, 1.07) [-57%]	1.01 (0.97, 1.06)	1.02 (0.97, 1.06)
3 [-0.01 (-0.25, 0.37)]	1.01 (0.97, 1.06)	1.01 (0.95, 1.08)	1.00 (0.94, 1.07)	1.02 (0.95, 1.08)
4 [0.94 (0.37, 14.31)]	1.03 (0.97, 1.08)	1.05 (0.97, 1.13) [78%]	1.04 (0.97, 1.12) [-15%]	1.05 (0.98, 1.13) [4%]
Intensity (public facilities) score				
1 [-0.75 (-1.25, -0.67)]	1	1	1	1
2 [-0.59 (-0.67, -0.40)]	1.01 (0.97, 1.05)	0.98 (0.94, 1.03)	0.98 (0.94, 1.03)	0.99 (0.94, 1.03)
3 [-0.06 (-0.40, 0.41)]	0.95 (0.91, 1.00)*	0.92 (0.86, 0.98)* [66%]	0.92 (0.86, 0.99)* [-8%]	0.93 (0.86, 0.99)* [-10%]
4 [1.14 (0.41, 10.26)]	1.03 (0.98, 1.09)	1.01 (0.94, 1.08)	1.02 (0.95, 1.09)	1.03 (0.96, 1.10)
Street Connectivity (alpha)				
1 [0.17 (-8.00, 0.21)]	1	1	1	1
2 [0.26 (0.21, 0.30)]	0.98 (0.93, 1.04)	0.98 (0.93, 1.03)	0.99 (0.94, 1.04)	0.99 (0.94, 1.04)
3 [0.33 (0.30, 0.38)]	0.96 (0.90, 1.01)	0.96 (0.91, 1.01) [-0%]	0.97 (0.92, 1.02) [-32%]	0.97 (0.93, 1.02) [-39%]
4 [0.45 (0.38, 8.00)]	0.95 (0.90, 1.01)	0.98 (0.93, 1.04) [-56%]	1.00 (0.94, 1.05)	1.00 (0.95, 1.05)
Advantageous socioeconomic/economic environment score^3^				
1 [-1.07 (-2.65, -0.71)]			1	1
2 [-0.30 (-0.70, 0.02)]			1.01 (0.96, 1.07)	1.01 (0.95, 1.07)
3 [0.22 (0.02, 0.55)]			1.04 (0.99, 1.10)	1.03 (0.97, 1.09)
4 [0.89 (0.55, 5.32)]			1.07 (1.01, 1.14)*	1.06 (0.98, 1.14)
Disadvantageous social environment score				
1 [-0.88 (-1.36, -0.61)]				1
2 [-0.36 (-0.61, -0.15)]				0.96 (0.92, 1.01)
3 [0.12 (-0.14, 0.43)]				0.98 (0.93, 1.04)
4 [1.15 (0.44, 4.11)]				0.95 (0.88, 1.02)

*Statistically significant (p < 0.05)

^1^National Longitudinal Study of Adolescent Health, Wave I (1995-96). Based on sex-stratified negative binomial regression models; value represents proportion increase in MVPA bouts. Referent category is lowest quartile.

^2^Change in coefficient reflects change in coefficient [(current model -previous model)/previous model]*100 for built environment characteristics only. Model 4 coefficients are compared to Model 2 coefficients. Change in estimates were omitted if both coefficients were <±0.04. Negative percent changes indicate attenuation of the association.

^3^In Model 3, denotes the 1-dimensional neighborhood SES factor; in model 4, denotes the Advantageous economic environment factor of the 2-dimensional neighborhood SES solution. Ranges for 1-dimensional factor quartiles: (1) -1.12 (-3.85, -0.64); (2) -0.13 (-0.64, 0.17); (3) 0.42 (0.17, 0.56); (4) 0.89 (0.56, 4.09)

Model 1: Built environment characteristics separately, adjusted for individual-level sociodemographics (age, race, parental education, household income, region)

Model 2: Built environment characteristics in the same model, adjusted for individual-level sociodemographics

Model 3: Built environment characteristics in the same model, adjusted for individual-level sociodemographics and 1-dimensional neighborhood SES factor

Model 4: Built environment characteristics in the same model, adjusted for individual-level sociodemographics and for 2-dimensional neighborhood SES factor

**Table 7:** Assessment of confounding to associations between built and socioeconomic environment factor score quartiles and weekly bouts of MVPA [exp(coeff)]^1^, Females (n = 8,626)

	**exp(coefficient) (95% CI) [change in coefficient**^**2**^**]**			
Quartile [median (range)]	Model 1	Model 2	Model 3	Model 4
Homogenous landscape score				
1 [-0.86 (-1.43, -0.68)]	1	1	1	1
2 [-0.49 (-0.68, -0.27)]	1.03 (0.98, 1.09)	1.04 (0.99, 1.09)	1.04 (0.98, 1.09)	1.03 (0.98, 1.09)
3 [0.00 (-0.27, 0.35)]	1.00 (0.96, 1.05)	1.02 (0.98, 1.07)	1.02 (0.97, 1.07)	1.02 (0.97, 1.06)
4 [1.04 (0.35, 5.46)]	1.00 (0.95, 1.05)	1.03 (0.97, 1.09)	1.03 (0.98, 1.10)	1.03 (0.97, 1.09)
Intensity (pay facilities) score				
1 [-0.82 (-1.45, -0.68)]	1	1	1	1
2[-0.51 (-0.68, -0.25)]	0.98 (0.94, 1.03)	0.97 (0.92, 1.02)	0.97 (0.91, 1.02)	0.97 (0.92, 1.03) [-11%]
3 [-0.01 (-0.25, 0.37)]	1.01 (0.96, 1.07)	0.99 (0.93, 1.05)	0.98 (0.93, 1.04)	0.99 (0.93, 1.05)
4 [0.94 (0.37, 14.31)]	0.93 (0.88, 0.99)*	0.91 (0.85, 0.98)* [33%]	0.91 (0.84, 0.98)* [4%]	0.92 (0.85, 0.99)* [-8%]
Intensity (public facilities) score				
1 [-0.75 (-1.25, -0.67)]	1	1	1	1
2 [-0.59 (-0.67, -0.40)]	0.97 (0.91, 1.03)	0.96 (0.91, 1.02) [17%]	0.97 (0.91, 1.03)	0.97 (0.92, 1.03) [-23%]
3 [-0.06 (-0.40, 0.41)]	0.99 (0.93, 1.05)	1.02 (0.95, 1.09)	1.02 (0.96, 1.09)	1.04 (0.98, 1.11) [159%]
4 [1.14 (0.41, 10.26)]	0.96 (0.90, 1.03)	1.02 (0.95, 1.10)	1.03 (0.96, 1.12)	1.06 (0.98, 1.14) [173%]
Street Connectivity (alpha)				
1 [0.17 (-8.00, 0.21)]	1	1	1	1
2 [0.26 (0.21, 0.30)]	0.98 (0.92, 1.05)	0.98 (0.92, 1.05)	0.99 (0.92, 1.06)	0.99 (0.92, 1.06)
3 [0.33 (0.30, 0.38)]	0.99 (0.93, 1.05)	0.99 (0.93, 1.06)	1.00 (0.94, 1.07)	1.01 (0.94, 1.07)
4 [0.45 (0.38, 8.00)]	0.92 (0.86, 0.98)*	0.90 (0.84, 0.97)* [15%]	0.92 (0.85, 0.99)* [-14%]	0.92 (0.86, 0.99)* [-22%]
Advantageous socioeconomic/economic environment score^3^				
1 [-1.07 (-2.65, -0.71)]			1	1
2 [-0.30 (-0.70, 0.02)]			1.03 (0.97, 1.08)	0.99 (0.93, 1.04)
3 [0.22 (0.02, 0.55)]			1.07 (1.00, 1.14)	1.02 (0.96, 1.09)
4 [0.89 (0.55, 5.32)]			1.06 (0.99, 1.14)	1.02 (0.96, 1.09)
Disadvantageous social environment score				
1 [-0.88 (-1.36, -0.61)]				1
2 [-0.36 (-0.61, -0.15)]				0.99 (0.94, 1.04)
3 [0.12 (-0.14, 0.43)]				0.91 (0.86, 0.97)*
4 [1.15 (0.44, 4.11)]				0.91 (0.84, 0.98)*

^1^National Longitudinal Study of Adolescent Health, Wave I (1995-96). Based on sex-stratified negative binomial regression models; value represents proportion increase in MVPA bouts. Referent category is lowest quartile.

*Statistically significant (p < 0.05)

^2^Change in coefficient reflects change in coefficient [(current model -previous model)/previous model]*100 for built environment characteristics only. Model 4 coefficients are compared to Model 2 coefficients. Change in estimates were omitted if both coefficients were <±0.04. Negative percent changes indicate attenuation of the association.

^3^In Model 3, denotes the 1-dimensional neighborhood SES factor; in model 4, denotes the Advantageous economic environment factor of the 2-dimensional neighborhood SES solution. Ranges for 1-dimensional factor quartiles: (1) -1.12 (-3.85, -0.64); (2) -0.13 (-0.64, 0.17); (3) 0.42 (0.17, 0.56); (4) 0.89 (0.56, 4.09)

Model 1: Built environment characteristics separately, adjusted for individual-level sociodemographics (age, race, parental education, household income, region)

Model 2: Built environment characteristics in the same model, adjusted for individual-level sociodemographics

Model 3: Built environment characteristics in the same model, adjusted for individual-level sociodemographics and 1-dimensional neighborhood SES factor

Model 4: Built environment characteristics in the same model, adjusted for individual-level sociodemographics and for 2-dimensional neighborhood SES factor

SES environment factors were also related to MVPA, with up to 7% higher MVPA for the highest versus lowest SES factor quartile in fully adjusted models (Tables 6 &7, Models 3 & 4). Comparison of Model 2 to Models 3 and 4 indicated confounding of MVPA associations with alpha and, in females, intensity (public facilities) by SES environment measures. The 2-dimensional SES factor (Model 3) influenced these associations to a greater extent than the 1-dimensional SES factor (Model 4), although absolute changes in estimates were small. The significant built environment-MVPA associations were otherwise relatively robust.

### MVPA and built and SES environment *single measures*: setting the stage for longitudinal settings and external study populations

Because factors are data-driven and population specific, we used knowledge gained from factor analysis to identify measures replicable in future research. Associations between MVPA and representative indicator measures (selected as per Methods, and flagged in Tables 3 &4) in Tables 8 &9 (Models 2 & 4) are generally consistent with corresponding factor score-MVPA associations, suggesting that the single measures adequately represent the underlying construct.

**Table 8:** Association between representative built, social, and economic environment measure quartiles and weekly bouts of MVPA, Males (n = 8,668)^1^

	exp(coefficient) (95% CI)		
Quartile [median (min, max)]	Model 2	Model 4	Model 5
Simpson's Diversity Index^2^			
1 [0.71 (0.66, 0.83)]	1	1	1
2 [0.62 (0.58, 0.66)]	0.96 (0.91, 1.01)	0.96 (0.91, 1.01)	0.96 (0.92, 1.01)
3 [0.53 (0.46, 0.58)]	0.91 (0.87, 0.94)*	0.91 (0.87, 0.95)*	0.91 (0.88, 0.95)*
4 [0.33 (0.01, 0.46)]	0.90 (0.86, 0.95)*	0.90 (0.86, 0.95)*	0.91 (0.86, 0.95)*
Count of pay facilities			
1 [0 (0, 1)]	1	1	1
2 [3 (2, 4)]	1.02 (0.98, 1.07)	1.02 (0.98, 1.06)	1.04 (0.97, 1.11)
3 [7 (5, 9)]	1.02 (0.97, 1.07)	1.02 (0.97, 1.07)	0.99 (0.94, 1.04)
4 [14 (10, 174)]	1.04 (0.99, 1.10)	1.05 (0.99, 1.10)	1.03 (0.99, 1.08)
Count of public facilities^3^			
1 [0 (0, 0)]	1	1	1
2 [1 (1, 1)]	0.96 (0.91, 1.02)	0.96 (0.91, 1.02)	1.05 (0.99, 1.12)
3 [3 (2, 20)]	1.03 (0.98, 1.07)	1.04 (1.00, 1.09)	1.01 (0.96, 1.05)
Street Connectivity (alpha)			
1 [0.17 (-8.00, 0.21)]	1	1	1
2 [0.26 (0.21, 0.30)]	0.97 (0.92, 1.03)	0.98 (0.93, 1.03)	0.98 (0.94, 1.03)
3 [0.33 (0.30, 0.38)]	0.95 (0.90, 1.00)	0.97 (0.92, 1.02)	0.97 (0.92, 1.02)
4 [0.45 (0.38, 8.00)]	0.98 (0.92, 1.04)	1.00 (0.94, 1.06)	1.01 (0.95, 1.06)
Median household income^4^			
1 [1.7 (0.5, 2.1)]		1	1
2 [2.5 (2.1, 3.0)]		1.01 (0.96, 1.07)	1.01 (0.97, 1.07)
3 [3.4 (3.0, 3.8)]		1.03 (0.98, 1.08)	1.03 (0.98, 1.08)
4 [4.5 (3.8, 12.5)]		1.08 (1.01, 1.16)*	1.08 (1.01, 1.15)*
Crime rate/100,000 population			
1 [2,629 (108, 3,647)]		1	1
2 [4,899 (3,696, 5,612)]		0.95 (0.90, 1.01)	0.96 (0.91, 1.02)
3 [6,177 (5,623, 6,975)]		0.94 (0.89, 1.00)*	0.96 (0.91, 1.02)
4 [8,317 (7,084, 13,723)]		0.96 (0.91, 1.01)	0.95 (0.91, 1.00)

^1^National Longitudinal Study of Adolescent Health, Wave I (1995-96). Based on sex-stratified negative binomial regression models; value represents proportion increase in MVPA bouts. Referent category is lowest quantile. Environmental measures representing each factor were generally selected based on the highest loadings, with the following exceptions: non-overlapping pay facility types (instruction, member, and public fee) were summed, public (rather than youth) facilities were selected for longitudinal relevance. For brevity, only Models 2, 4, and 5 are presented; their names are retained to be consistent with **Table 6**.

^2^Negatively associated with homogenous land cover factor, so reverse coded to for comparability

^3^tertiles

^4^in 10,000's

Model 2: Built environment characteristics in the same model, adjusted for individual-level sociodemographic variables

Model 4: Built environment characteristics in the same model, adjusted for individual-level sociodemographics, median household income, and crime rate

Model 5: Built environment characteristics (facilities counts scaled by population) in the same model, adjusted for individual-level sociodemographics, median household income, and crime rate

*Statistically significant (p < 0.05)

**Table 9:** Association between representative built, social, and economic environment measure quartiles and weekly bouts of MVPA, Females (n = 8,626)^1^

	exp(coefficient) (95% CI)		
Quartile [median (min, max)]	Model 2	Model 4	Model 5
Simpson's Diversity Index^2^			
1 [0.71 (0.66, 0.83)]	1	1	1
2 [0.62 (0.58, 0.66)]	1.04 (0.98, 1.10)	1.03 (0.97, 1.10)	1.03 (0.97, 1.10)
3 [0.53 (0.46, 0.58)]	1.04 (0.99, 1.10)	1.04 (0.99, 1.10)	1.03 (0.98, 1.09)
4 [0.33 (0.01, 0.46)]	1.00 (0.95, 1.06)	1.01 (0.95, 1.06)	1.00 (0.94, 1.05)
Count of pay facilities			
1 [0 (0, 1)]	1	1	1
2 [3 (2, 4)]	0.97 (0.92, 1.02)	0.96 (0.92, 1.01)	0.93 (0.88, 0.99)*
3 [7 (5, 9)]	0.97 (0.92, 1.02)	0.96 (0.92, 1.01)	0.95 (0.90, 0.99)*
4 [14 (10, 174)]	0.92 (0.86, 0.98)*	0.92 (0.87, 0.98)*	0.96 (0.91, 1.01)
Count of public facilities^3^			
1 [0 (0, 0)]	1	1	1
2 [1 (1, 1)]	1.03 (0.97, 1.09)	1.04 (0.99, 1.09)	1.04 (0.95, 1.13)
3 [3 (2, 20)]	1.05 (0.99, 1.11)	1.07 (1.02, 1.13)*	1.04 (1.00, 1.09)*
Street Connectivity (alpha)			
1 [0.17 (-8.00, 0.21)]	1	1	1
2 [0.26 (0.21, 0.30)]	0.99 (0.93, 1.06)	1.00 (0.94, 1.07)	0.99 (0.93, 1.06)
3 [0.33 (0.30, 0.38)]	0.99 (0.93, 1.06)	1.02 (0.96, 1.09)	1.02 (0.95, 1.09)
4 [0.45 (0.38, 8.00)]	0.91 (0.85, 0.98)*	0.94 (0.87, 1.01)	0.94 (0.87, 1.01)
Median household income^4^			
1 [1.7 (0.5, 2.1)]		1	1
2 [2.5 (2.1, 3.0)]		1.03 (0.98, 1.08)	1.03 (0.98, 1.08)
3 [3.4 (3.0, 3.8)]		1.05 (0.99, 1.12)	1.05 (0.99, 1.12)
4 [4.5 (3.8, 12.5)]		1.10 (1.02, 1.18)*	1.11 (1.03, 1.19)*
Crime rate/100,000 population			
1 [2,629 (108, 3,647)]		1	1
2 [4,899 (3,696, 5,612)]		0.98 (0.92, 1.04)	0.98 (0.93, 1.04)
3 [6,177 (5,623, 6,975)]		0.93 (0.86, 1.00)*	0.94 (0.87, 1.01)
4 [8,317 (7,084, 13,723)]		0.92 (0.86, 0.98)*	0.93 (0.87, 1.00)*

^1^National Longitudinal Study of Adolescent Health, Wave I (1995-96). Based on sex-stratified negative binomial regression models; value represents proportion increase in MVPA bouts. Referent category is lowest quantile. Environmental measures representing each factor were generally selected based on the highest loadings, with the following exceptions: non-overlapping pay facility types (instruction, member, and public fee) were summed, public (rather than youth) facilities were selected for longitudinal relevance. For brevity, only Models 2, 4, and 5 are presented; their names are retained to be consistent with **Table 7**.

^2^Negatively associated with homogenous land cover factor, so reverse coded to for comparability

^3^tertiles

^4^in 10,000's

Model 2: Built environment characteristics in the same model, adjusted for individual-level sociodemographic variables

Model 4: Built environment characteristics in the same model, adjusted for individual-level sociodemographics, median household income, and crime rate

Model 5: Built environment characteristics (facilities counts scaled by population) in the same model, adjusted for individual-level sociodemographics, median household income, and crime rate

*Statistically significant (p < 0.05)

The emergence of "intensity" factors suggests that facility counts may reflect a general density of development and resources. Model 5 used alternative facilities variables scaled by population, either attenuating or magnifying facilities-MVPA associations.

## Discussion

Neighborhood environments that may encourage or discourage physical activity are complex and multidimensional, but most existing research examines single or only a few aspects of the environment. Our study shows inter-relatedness of environmental characteristics in a nationally representative adolescent population and reveals several patterns of built and SES environments reflecting constructs consistent with research in adult populations. Further, correlations among environment characteristics resulted in confounding to estimated associations with MVPA, demonstrating the complexity of potential environmental influences on physical activity.

### Insights about the environment gained from pattern analysis

Our factor analysis identified inter-relationships among environmental measures too tightly correlated to analyze simultaneously as individual measures, while less inter-correlated environmental characteristics can be analyzed using traditional multivariate methods.

#### Inseparability of environmental features

In existing research, single environment measures are often examined as indicators of isolated environment characteristics. For example, intersection density is a common measure of street connectivity [18,34-36], and facilities counts are often used to indicate access to resources. However, dense, gridded streets are common in city centers [37], which represent a multitude of built, socioeconomic, and other features, and it is intuitive that more physical activity facilities are located in otherwise densely developed areas. Indeed, Cervero and colleagues [16] introduced the concept of intensity, representing dense population and resources and interpreted as a measure of density. Consistent with this conceptualization, our study demonstrated that counts of physical activity facilities were strongly linked with population and intersection density, suggesting that it is important to adjust for density in estimation of physical activity facilities' effects. Yet statistical adjustment may be inappropriate due to strong correlation between density measures and facilities counts. Instead, we found that ratios of physical activity facilities per 1,000 population was a useful strategy for separating density from count of facilities, similar to Diez Roux and colleagues [2].

In contrast, other street connectivity measures did not load onto factors in our study, indicating that they were not strongly correlated with each other or with other aspects of the built environment. Our results contrast with other studies showing constructs with multiple connectivity index indicators [12,14]. This discrepancy may be explained by the national scope of Add Health as opposed to one or more metropolitan areas in the studies noted. Connectivity indices are ratios of various components such as number of intersections, street segments, and route alternatives, so they may reflect different constructs in areas with high versus low component values. Likewise, Ewing et al [17] reported a single principal component representing urban sprawl characterized by residential density, land use mix, and street accessibility in a national sample, but their study was also limited to metropolitan areas and used block size measures rather than connectivity indices to represent street accessibility. Alternatively, our buffer-defined areas may influence intersection and street segment counts, particularly in rural areas with few streets, altering the meaning of the connectivity indices.

#### Dimensionality of environmental constructs

Factor solutions distinguished dimensions of similar constructs, which in turn were differentially related to MVPA. Factor analysis identified two types of facilities which were related to MVPA in different ways. For example, in females, MVPA was negatively associated with intensity (pay facilities) but marginally positively associated with intensity (public facilities) in fully adjusted models. Likewise, two SES environment factors emerged, one reflecting economic and education characteristics, the other reflecting social characteristics. These factors were correlated but appear to be differentially related to the built environment and MVPA.

### Importance of incorporating many aspects of the environment when estimating neighborhood effects on physical activity

Factors allowed a wide range of environmental measures to be simultaneously incorporated into the analysis, revealing confounding by SES and built environment characteristics:

#### Confounding by SES environment characteristics

In particular, built and SES factors were strongly associated, and adjustment for SES environment factor(s) resulted in changes to several built environment-MVPA associations. Further, the 2-dimensional SES environment construct was a stronger confounder of associations between MVPA and intensity (public facilities) and, to a lesser extent, street connectivity, compared to the 1-dimensional construct. Such confounding could reflect placement of public facilities in areas of greatest need. Likewise, high street connectedness is common in poor inner-city areas where physical activity may be influenced by social contexts particularly relevant to females such as crime [38], which is better captured by the 2-dimensional SES environment construct.

These results support our conceptualization of the SES environment as a confounder of the built environment-MVPA association. However, relationships between the built and SES environments may be bidirectional and dynamic. For example, crime may mediate, rather than confound, relationships between built and SES environment measures and physical activity. Furthermore, the social and economic resources of a community may influence where built environment features are situated, social norms with regard to health behavior [39], and perceived and objective safety; ultimately, the SES environment measures may be surrogates for a multitude of influences on MVPA. While future research should investigate and account for these complexities, examination of the SES and built environments as independent influences on MVPA is valuable for documenting SES disparities and investigating the potential benefits of modifying the built environment while accounting for inter-correlation with the less modifiable SES environment.

#### Confounding by built environment characteristics

While built environment characteristics met our objective definition of confounding, absolute changes to estimates were small and did not change study conclusions regarding the relationship between the built environment and MVPA. One possible explanation for weak confounding is that our built environment factors were multidimensional and account for correlations between built environment measures; individual built environment measures may confound other measures loading onto the same factor. However, strong correlations preclude formal testing of this hypothesis. Additionally, the degree of confounding by built and SES environment characteristics in our study may have been minimized by weak built environment-MVPA relationships.

#### Implications

These findings suggest that failure to adjust for both economic and social aspects of the SES environment may lead to biased estimates of some built environment-MVPA associations. Fortunately, census variables are readily available. In contrast, relatively weak confounding by other built environment characteristics is encouraging for studies without the wide range of measures used in this study. However, in several cases, simultaneously adjusting for multiple built environment measures *magnified *the associations. Furthermore, in studies showing stronger associations or examining one-dimensional built environment measures, omission of additional built environment characteristics may lead to more substantial underestimation of effects. Finally, even small degrees of confounding may influence conclusions drawn from generally weak associations in the extant literature.

### Forging ahead with replicable measures into longitudinal settings and external populations

Multidimensional built and SES environment constructs identified from factor analysis allowed us to simultaneously examine a large set of measures with respect to MVPA. In a next step, we used the knowledge gained from factor analysis to create simplified measures (Tables 8 &9) that incorporate inter-relationships, yet are more easily replicable in future studies. We emphasize that our simplified measures represent the set of variables identified using factor analysis and should be interpreted as such. In fact, replication of regression results with single indicators demonstrates that these measures, which are often analyzed on their own, may act as proxies for underlying environmental constructs.

Two branches of investigation are needed to better understand the potential causal effects of these measures. First, these simplified measures can be used in longitudinal analyses and examination in external populations. As opposed to other strategies such as scale measures, they are readily understandable and examined in prior research, and selection of single indicators reduces the number of measures needed to replicate findings in other studies.

Second, investigation of mechanisms leading to the observed associations will help to distinguish between proxies and policy-relevant determinants of physical activity. For example, crime replicated associations between the disadvantageous social environment factor and MVPA, but how crime might influence physical activity, or if yet another characteristic is the causal agent, is unknown. Research incorporating psychological measures (e.g., self-efficacy and perceived barriers) or detailed audit-based environment data (e.g., aesthetics and quality of facilities) can improve understanding of behavioral mechanisms. Such research may reveal additional layers, possibly showing our multidimensional environment constructs as proxies for more qualitative inter-personal or cultural aspects of the environment.

Determining whether patterning of environmental measures is similar in other populations is an important next step. If patterning in other age groups differs substantially from our nationally representative sample of adolescents, our simple measures may have limited ability to represent the constructs identified in this study and thus must be tested before applying them in other populations.

We found differences in built environment-MVPA associations by sex, which is consistent with previous studies examining walkability and physical activity resources [32,40,41]. Homogenous landscape appears to be a negative correlate of MVPA for males but not females, possibly because males may be more likely to be active outdoors [42] with less regard to safety or other concerns. Intensity (pay facilities) was associated with lower MVPA in females but not males. On the other hand, count of public facilities corrected for population was associated with higher MVPA in females but not males, perhaps also due to safety concerns addressed by access to facilities. Such differences by sex may shift as adolescents age into adulthood, when overall physical activity levels are lower [19], or decrease among adolescents over time as physical activity promotion efforts in recent decades may have addressed barriers such as safety or provided additional sex-neutral activity opportunities.

Further investigation of the dose-response relationship between the built environment and MVPA is another opportunity for future research. We found non-linear associations between four aspects of the built environment and MVPA. The strongest associations were generally observed for the largest quartile, which, due to data skewness, contained very large factor score or measure values. Using quartile measures allowed comparability between associations with factors versus single indicators, but closer examination of dose-response and shape of the relationship is warranted. Shifts in the shape of the dose-response relationships - sometimes alternating between monotonic and U-shaped - with additional covariates add complexity and should be further examined.

### Limitations and Strengths

Limitations include cross-sectional study design, which does not imply causality. Yet, we identified replicable measures that set the stage for longitudinal analyses, which can establish temporality and better address bias due to residential self-selection [43,44]. Second, there was some temporal mismatch between individual-level interviews (1995-96) and GIS data sources (e.g., StreetMap 2000, 1992 land cover dataset), but our GIS is unique in providing historical data approximately contemporaneous with multiple survey waves. Our county-level crime measure was crude, yet it provided an objective measure of safety available across the US that was strongly associated with MVPA. Third, while we analyzed an extensive number of environmental variables, we did not consider quality of facilities, perceived environment measures, or other potential psychological mediators.

Fourth, we examined overall leisure time MVPA frequency, which does not distinguish between possible behavior-specific effects [45] or incorporate physical activity duration or intensity. Our built and SES environment measures may show stronger relationships with specific types of physical activity. For example, stronger relationships may be present between alpha street connectivity and active transportation behaviors, or between pay or public facilities and team sports or exercise. Clearly, future research should examine behavior-specific associations while accounting for complex patterning of the environment.

Finally, we did not address urbanicity, which may be an important moderator [46] of built environment-MVPA relationships. However, our study informs a growing body of work using national datasets by addressing environment patterning and confounding in broad range of neighborhood environments as well as examining measures applicable longitudinally during periods in which individuals may move in or out of urban areas. Additionally, the wide range of existing urbanicity measures are generally based on environment characteristics of interest (e.g., population density), thereby obscuring practical applications such as modification of the built environment in suburban areas to more closely resemble urban areas. Nevertheless, analogous analysis stratified by some measure of urbanicity is an important next step.

Additional strengths include examination of a wide range of environment measures in a nationally representative sample of adolescents, an understudied population. We explicitly examined and compared built and SES environment characteristics, which were strongly related. Finally, we used pattern analysis methods to not only investigate inter-relationships, but also to inform the creation of replicable measures.

## Conclusions

Our study demonstrates substantial inter-relationships between environmental characteristics and suggests that many aspects of the built and SES environments should be incorporated into analysis in order to minimize confounding. Further, commonly used built environment measures may reflect more general environmental patterning and should be interpreted as such. Examination of how a broad range of environmental characteristics mutually influenced their relationships with physical activity suggested complex mechanisms involving a myriad of social and cultural factors. Finally, we present simplified, replicable measures that are cross-sectionally related to physical activity in adolescents. Better characterization of the environment, longitudinal analysis, and exploration of mechanisms in future studies can increase our understanding of built environment features that should be targeted in physical activity promotion policy.

## Competing interests

There were no potential or real conflicts of financial or personal interest with the financial sponsors of the scientific project.

## Authors' contributions

JBH, KRE, YS, and PGL contributed to the study design, JBH and PGL contributed to the data analysis. JBH drafted the manuscript; all four authors provided critical feedback and approved the manuscript.

## Supplementary Material

Additional file 1 **Appendix**. Table A1. Individual-level characteristics by sex Click here for file
