# Supplementary material for: Built and socioeconomic environments: patterning and associations with physical activity in U.S. adolescents
Source: Int J Behav Nutr Phys Act. 2010 May 20;7:45. doi: 10.1186/1479-5868-7-45 (PMC3152773; doi:10.1186/1479-5868-7-45)
Supplement: Additional file 1 — Appendix. Table A1. Individual-level characteristics by sex [file 1479-5868-7-45-S1.DOC]

**Appendix**

**Table A1.** Individual-level characteristics by sex [mean/% (SE)]1

|  | Males | Females |
| --- | --- | --- |
| Count | 8,668 | 8,626 |
| Race/ethnicity (%) |  |  |
| White | 68.0 (2.9) | 68.3 (3.0) |
| Black | 16.0 (2.1) | 16.0 (2.1) |
| Asian | 3.5 (0.7) | 3.4 (0.7) |
| Hispanic | 12.4 (1.8) | 12.3 (1.8) |
| Parent education (%) |  |  |
| <High school | 15.0 (1.4) | 15.7 (1.4) |
| High school/GED | 32.2 (1.2) | 33.1 (1.2) |
| Some college | 28.5 (1.0) | 27.1 (0.9) |
| College or greater | 24.3 (1.6) | 24.1 (1.6) |
| Region (%) |  |  |
| West | 14.9 (1.4) | 15.7 (1.4) |
| Midwest | 31.3 (2.3) | 32.7 (2.6) |
| South | 38.9 (1.8) | 37.9 (1.9) |
| Northeast | 14.9 (1.0) | 13.7 (0.9) |
| Age (mean) | 15.5 (0.1) | 15.3 (0.1) |
| MVPA (mean # weekly bouts) | 7.2 (0.1) | 5.7 (0.1) |
| Household income (mean) | 43.0 (1.5) | 43.3 (1.5) |

^1^ National Longitudinal Study of Adolescent Health, Wave I (1995-96), n=17,296.
